# Decoding the Endometriosis-Associated Infertility Microenvironment: A Review of FTIR and Raman Spectroscopic Insights into Follicular Fluid

**DOI:** 10.3390/cimb48030303

**Published:** 2026-03-12

**Authors:** Piotr Olcha, Igor Hawryluk, Joanna Depciuch

**Affiliations:** 1 Department of Gynecology and Gynecological Endocrinology, Medical University of Lublin, Aleje Racławickie 23, 20-049 Lublin, Poland; 2 In Vitro Infertility Treatment Center “Gravida”, Aleja Armii Krajowej 21, 09-409 Plock, Poland; hawryluk.igor@gmail.com; 3 Institute of Nuclear Physics, Polish Academy of Science, Radzikowskiego 152, 31-342 Krakow, Poland

**Keywords:** endometriosis, follicular fluid, Fourier-Transform Infrared (FTIR) spectroscopy, Raman spectroscopy, infertility, oocyte quality

## Abstract

**Background**: Endometriosis is a major cause of female infertility. It significantly impacts oocyte quality and embryonic development. The condition’s pathophysiological mechanisms are multifactorial. However, they are believed to be reflected in the biochemical composition of follicular fluid (FF). FF is the immediate microenvironment of the developing oocyte hence its relevance. Conventional analytical methods provide only a limited view of this complex biofluid. This underlies the need for holistic profiling techniques. **Objective**: This narrative review synthesizes current knowledge on the potential of Fourier-Transform Infrared (FTIR) and Raman spectroscopy. The two are scrutinized as label-free, non-destructive tools for analyzing FF in the context of endometriosis. As such, the aim is to bridge the understanding of the disease’s impact on the follicular niche with the analytical power of these spectroscopic techniques, ultimately highlighting a critical research gap, while critically evaluating the translational pathway required to bring these techniques from research laboratories into routine clinical IVF practice. This includes assessment of practical feasibility, cost-effectiveness, turnaround time, standardization requirements, and comparison with existing clinical biomarkers. **Methods**: We outline the fundamental principles of FTIR and Raman spectroscopy and their complementary strengths. The review then consolidates evidence from proteomic and metabolomic studies demonstrating FF alterations in endometriosis. We also showcase the successful application of vibrational spectroscopy in other reproductive diagnostics. This synthesis is vital to identifying a specific unmet need in the field. **Conclusions**: Despite the known importance of FF and the proven capability of FTIR and Raman spectroscopy in related areas, there is a striking lack of studies applying these techniques directly to the FF of women with endometriosis. This review concludes by framing this void as a pivotal research opportunity. In doing so, it presents a direct rationale and methodological framework for a future study designed to characterize the unique spectral fingerprints of endometriosis in FF, with the goal of uncovering novel biomarkers and pathophysiological insights.

## 1. Introduction

This narrative review is based on a comprehensive survey of the peer-reviewed literature. Relevant publications were identified primarily through searches of the PubMed database. Keywords included combinations of terms such as FTIR, Raman spectroscopy, vibrational spectroscopy, reproductive medicine, follicular fluid, oocyte, endometrium, biofluids, and in vitro/in vivo studies. Additional articles were identified through manual screening of reference lists from relevant publications. The selection of articles was guided by their relevance to the application of FTIR and Raman spectroscopy in reproductive biology and medicine, with an emphasis on methodological approaches, spectral interpretation, and biological context rather than exhaustive quantitative comparison. As this is a narrative review, no formal inclusion or exclusion criteria were applied.

### 1.1. The Clinical Puzzle: Endometriosis and Infertility

#### 1.1.1. Brief Epidemiology of Endometriosis

Endometriosis is a common, estrogen-dependent, chronic inflammatory gynecological condition defined by the presence of endometrial-like tissue outside the uterine cavity [1]. It is primarily a disease of the reproductive years, with a peak incidence diagnosed in women aged 25 to 35 years [2]. The reported prevalence varies considerably depending on the population studied. Within the general population of reproductive-aged women, approximately 6–10% are affected [1,3]. This rate increases dramatically in specific subpopulations. For instance, it is found in 20–50% of infertile women and can be as high as 71–87% in women with chronic pelvic pain [1,2,3].

Endometriosis often presents with a significant diagnostic delay. Studies consistently show a mean delay of 6.7 years from symptom onset to surgical confirmation. This is a latency period that has remained largely unchanged for decades and contributes to reduced quality of life and disease progression [3,4].

Several risk factors have been identified. A consistent finding is an increased risk among women with a first-degree relative with the condition. This suggests a strong heritable component [1,2]. Other established risk factors include early menarche, shorter menstrual cycles, and heavier menstrual flow [2,3].

#### 1.1.2. The Strong Association Between Endometriosis and Infertility: Prevalence Statistics

The prevalence of endometriosis is markedly higher in infertile populations compared to the general population. Multiple clinical studies have demonstrated this disparity. In a study of infertile women in South India, the prevalence of endometriosis was found to be 30% [5]. Similarly, a one-year retrospective study reported that 33.69% of the infertile women in their cohort were diagnosed with endometriosis [6]. This high prevalence is particularly striking among women with specific infertility diagnoses. In a recent study focused on a specific cohort of patients with unexplained infertility who had failed prior fertility treatments, laparoscopic investigation with histological confirmation revealed a remarkably high prevalence of endometriosis at 90.7%. Contrary to the common perception that infertility is primarily linked to minimal or mild disease, the majority of patients in this study (65.0%) were diagnosed with moderate to severe endometriosis (Stage III or IV according to the rASRM classification) [7].

Conversely, infertility is a common consequence for women diagnosed with endometriosis. It is estimated that 30–50% of women with endometriosis are infertile [8,9]. This relationship appears to be causal. A 2024 Mendelian randomization study provided genetic evidence. The conclusions were that endometriosis has a causal effect on increasing the risk of both female infertility and primary ovarian failure [10].

The impact of endometriosis on fertility prospects is significant. Women with endometriosis have been shown to face a lower monthly probability of conception (fecundity) compared to women without the condition [11]. This association has been consistently observed over time. For instance, a decade-long trend study confirmed that endometriosis is the major contributor to infertility cases [12]. The pathophysiology is multifactorial. It involves a combination of anatomical distortion, chronic inflammation, impaired implantation, and reduced oocyte quality. All these collectively contribute to the subfertile state [9,13].

#### 1.1.3. Overview of Proposed Pathophysiological Mechanisms Linking Endometriosis to Infertility

##### Anatomical Distortion (Pelvic Adhesions, Tubal Dysfunction)

Anatomical distortion of the pelvic organs is the primary mechanical factor linking endometriosis to infertility. The chronic inflammatory process can lead to the formation of adhesions and scar tissue, which may distort pelvic anatomy, restrict organ mobility, and cause tubal dysfunction [13,14]. These adhesions tend to envelop the ovaries and fallopian tubes. As a result, this potentially leads to frank tubal occlusion or impaired fimbrial function, thus hindering ovum pick-up and transport [15,16]. The distorted pelvic environment physically disrupts the crucial interaction between the ovary, fallopian tube, and ovulated oocyte, presenting a significant barrier to natural conception [17].

#### 1.1.4. Chronic Inflammation and Oxidative Stress in the Pelvic Environment

**Figure 1:**
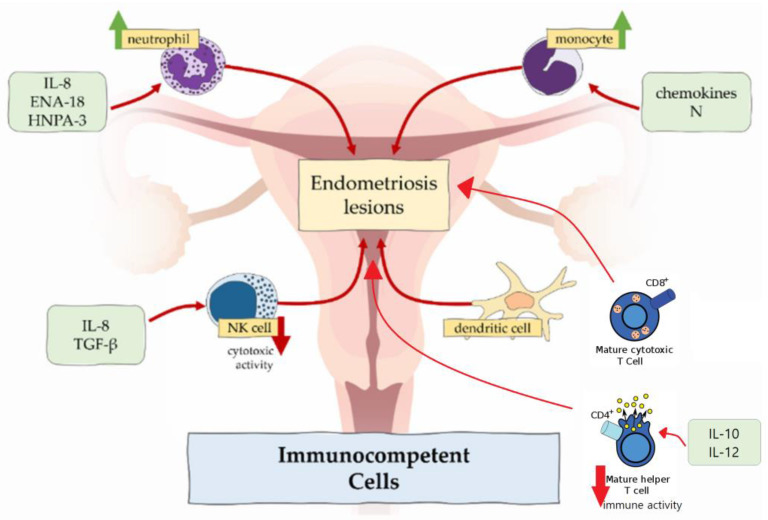
Reprinted from Lamceva et al., International Journal of Molecular Sciences, 2023, under CC BY 4.0 (https://creativecommons.org/licenses/by-nc/4.0/) [27]. This figure exhibits the immune cells that secrete the pro-inflammatory cytokines such as tumor necrosis factor-alpha (TNF-α) and interleukins (IL-1β, IL-6) [27]. These underlie a constant inflammation cycle that fosters oxidative stress.

A key aspect of endometriosis is the state of chronic inflammation and elevated oxidative stress within the pelvic peritoneal cavity. This creates a hostile microenvironment for reproduction. The condition is characterized by an influx of immune cells, including activated macrophages, which secrete pro-inflammatory cytokines such as tumor necrosis factor-alpha (TNF-α) and interleukins (IL-1β, IL-6) as seen in Figure 1, establishing a self-perpetuating inflammatory cycle [18,19]. This inflammatory milieu is intrinsically linked to oxidative stress. It is marked by an imbalance between elevated levels of reactive oxygen species (ROS) and a deficiency in antioxidant defenses [20,21]. The resulting oxidative damage has significant consequences. These include potential damage to sperm DNA, impairment of oocyte quality and maturation, and negative impacts on embryo development and implantation [22,23]. Further, this inflammatory-oxide state can alter the endometrial receptivity by disrupting the expression of adhesion molecules and the precise hormonal signaling necessary for successful embryo attachment [24,25]. Ultimately, this compromised pelvic and intrafollicular environment is a key contributor to endometriosis-associated infertility [13,26].

#### 1.1.5. Altered Hormonal Milieu and Impaired Folliculogenesis

Endometriosis disrupts hormonal balance necessary for normal ovarian function and oocyte development. Essentially, the follicular fluid of women with endometriosis exhibits a distinct hormonal profile [28]. It is characterized by elevated levels of estradiol and reduced bioavailability of progesterone. These hormonal alterations within the follicular fluid directly affect the cumulus–oocyte complex [29]. This is because steroid hormones can diffuse through cumulus cell layers and bind to receptors on both cumulus cells and the oocyte itself [30]. Additionally, altered gene expression within granulosa cells compromises their steroidogenic function and the gap junction-mediated communication essential for coordinated follicular growth [31,32]. The presence of endometriomas is particularly detrimental, as they can cause direct physical damage to the ovarian cortex, reduce ovarian reserve, and disrupt the pool of primordial follicles, which inevitably results in impaired folliculogenesis [33,34].

#### 1.1.6. Compromised Oocyte Quality and Embryonic Competence

Oocytes from women with endometriosis demonstrate morphological abnormalities, reduced fertilization rates, and impaired pre-implantation embryonic development [33,34,35]. This diminished oocyte competence is reflected in clinical outcomes. That is, endometriosis patients undergoing IVF exhibit lower numbers of high-quality embryos and reduced implantation and pregnancy rates compared to women with other causes of infertility [36,37]. The pathogenic basis for this is multifactorial. It mainly stems from the altered follicular environment, which exposes the developing oocyte to inflammatory cytokines and oxidative stress. The outcome is molecular and cellular damage [38,39]. This hostile microenvironment can induce mitochondrial dysfunction, cytoskeletal alterations, and DNA damage in the oocyte. The significant result from this is embryos with reduced developmental competence and a higher incidence of aneuploidy [9,40]. Therefore, the detrimental impact of endometriosis on the oocyte itself is the main cause for less successful pregnancies.

The collective evidence from the above mechanisms solidifies the central hypothesis that the follicular fluid, which is the oocyte’s immediate microenvironment, must reflect pertinent pathological changes. It is therefore relevant to analyze for specific biochemical signatures. This review focuses on the application of FTIR and Raman spectroscopy. The two are powerful analytical tools that are uniquely positioned to decode the potential spectral fingerprints and provide molecular insight into endometriosis-associated infertility.

## 2. Follicular Fluid: A Window into Oocyte Health: Definition, Origin, and Composition of Follicular Fluid

**Figure 2:**
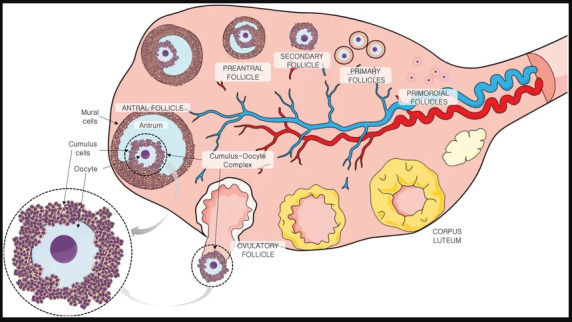
Reprinted from von Mengden et al. *Antioxidants and Redox Signaling*, 2020, under CC BY 4.0 (https://creativecommons.org/licenses/by-nc/4.0/) [50]. Anatomy of the ovary. In the mature antral follicle, the oocyte is surrounded by specialized granulosa cells. These are named the cumulus cells, which are in contact with the follicular fluid inside the antrum [50]. Our main focus is on the follicular fluid, which, as shown here, surrounds the oocyte. Rationale: This figure establishes foundational anatomical knowledge before introducing spectroscopic applications, helping spectroscopy specialists visualize the physical context of follicular fluid.

Follicular fluid (FF) is an important biological fluid that fills the antrum of developing ovarian follicles (The antrum is shown in Figure 2 below). As such, it is the immediate microenvironment for the oocyte [41,42]. FF originates from a dual process. First is the transudation of blood plasma components across the blood–follicle barrier. Second is the active secretory products from the surrounding granulosa and theca cells [43,44]. As a result, its composition is highly complex and dynamic. FF comprises a rich mixture of hormones, metabolites, proteins, antioxidants, electrolytes, and even cellular components like exosomes and epithelial cells [45,46,47,48]. This unique biochemical milieu varies with the stage of follicular development. The health and functional status of the follicle is the main influencer, making it a direct reflector of oocyte quality and developmental potential [42,49].

### 2.1. The Role of FF in Oocyte Maturation, Metabolism, and Protection

FF plays an important and active role in supporting the final stages of oocyte development. It provides the necessary signaling molecules and hormonal cues that drive the resumption of meiosis and nuclear maturation. This ensures that the oocyte becomes competent for fertilization [42,51]. Crucially, FF serves as a reservoir of metabolites that support the energy metabolism of both cumulus cells and the oocyte. It supplies substrates like pyruvate, lipids, and amino acids that are essential for ATP production and cytoplasmic maturation [52,53]. However, it is important to recognize that the oocyte itself has limited capacity for direct uptake of certain nutrients. Instead, cumulus cells internalize these substrates from FF and transfer processed metabolites, such as pyruvate converted from glucose, to the oocyte via gap junctions [52,54]. This metabolic cooperativity means that FF composition indirectly influences oocyte metabolism through its effects on cumulus cell function. Further, FF components offer vital protection. Extracellular vesicles within FF can deliver bioactive molecules, including miRNAs, proteins, and lipids, to cumulus cells and potentially directly to the oocyte. This supports cellular metabolism and improves embryo development [41,55]. Additionally, the fluid’s overall composition, including antioxidants, helps shield the fragile oocyte from oxidative stress. This is mainly by neutralizing reactive oxygen species that penetrate the cumulus cell layers [42,56]. Thus, FF functions both as a direct source of certain molecules and as an indirect regulator of oocyte health through its modulation of cumulus cell activity.

### 2.2. Rationale for Studying FF to Understand Oocyte Quality and Reproductive Outcomes

The rationale for studying FF is informed by its role as the immediate environment of the developing oocyte. The oocyte is avascular and exists within a complex support system that involves two complementary routes of molecular exchange: (1) direct transfer of small molecules, metabolites, and signaling factors from the surrounding cumulus cells via gap junctions, and (2) diffusion of components from the follicular fluid that bathes the cumulus–oocyte complex [51,52]. Cumulus cells extend transzonal projections through the zona pellucida to form gap junctions with the oolemma, enabling transfer of nutrients (such as pyruvate, amino acids, and nucleotides) and regulatory molecules that are essential for meiotic progression and cytoplasmic maturation [42,53]. Simultaneously, the follicular fluid provides the bulk extracellular milieu, supplying macromolecules, hormones, growth factors, and antioxidants that cannot be synthesized by the cumulus–oocyte complex itself [41,54]. These two support systems are intimately linked. Cumulus cells actively modify their local microenvironment and can take up components from follicular fluid for subsequent transfer to the oocyte. Therefore, while the oocyte does not rely exclusively on FF in a direct sense, FF composition fundamentally reflects the biochemical environment to which both cumulus cells and the oocyte are exposed. Further, it contains molecules that ultimately reach the oocyte through both direct diffusion and cumulus cell-mediated transport. Hence, analyzing FF provides a window into the cumulative nutritional, hormonal, and stress-related conditions that shape oocyte developmental competence.

### 2.3. The Analytical Challenge: Probing Complex Biofluids

Conventionally, there are targeted analytical methods, such as enzyme-linked immunosorbent assays (ELISA) for specific cytokines or standard hormone assays. While valuable, they are significantly limited in comprehensively characterizing a complex biofluid such as FF. These techniques are mainly hypothesis–driven. They require prior knowledge of specific analytes to measure, which only provides a narrow, pre-defined preview of the fluid’s composition. Hence, there is a pressing need for broad, untargeted “omics” approaches. The emerging field of spectranomics (vibrational spectroscopy-based omics) is one of them. Unlike conventional methods, these can simultaneously analyze a wide array of molecules without pre-selection. Therefore, these holistic strategies are essential to uncover the complex, multi-factorial biochemical signatures of conditions like endometriosis. The transition is moving beyond single biomarkers to a systems-level understanding of infertility.

### 2.4. FTIR and Raman Spectroscopy: Emerging Tools in Reproductive Bioanalysis

In response to the limitations of conventional targeted assays, Fourier-Transform Infrared and Raman spectroscopy have increasingly gained recognition as advanced, label-free, and non-destructive analytical platforms. Both methods rely on vibrational spectroscopy. Nevertheless, they differ in the nature of light–matter interactions they exploit: FTIR measures the absorption of infrared radiation by molecular bonds [55,56,57]. In contrast, Raman spectroscopy detects inelastic scattering of monochromatic laser light [58,59]. Despite these differences, their shared strength lies in their ability to generate a comprehensive “biochemical fingerprint” reflective of the molecular complexity of the analyzed material [58,60]. This analytical rationale is particularly compelling for FF analysis. FF is a dynamic microenvironment that reflects the metabolic, endocrine, and inflammatory status of the follicle. Compared to targeted techniques, FTIR and Raman spectroscopy can simultaneously detect, differentiate, and semi-quantify broad molecular categories. These include proteins, lipids, carbohydrates, nucleic acids, and various metabolites, from a single, minimally processed sample [56,57,61,62]. Such holistic profiling enables the identification of subtle biochemical alterations that are undetectable by conventional assays. Hence, it offers a more integrative understanding of the follicle’s functional state.

Importantly, these methods require no labeling. They also exert minimal sample manipulation and ensure sample integrity preservation [55,57,59]. This makes them particularly suitable for studying delicate biological matrices. Given the multifactorial nature of endometriosis-associated infertility, the ability of FTIR and Raman spectroscopy is crucial. They capture complex, multi-constituent spectral signatures that uncover pathophysiological patterns and potential diagnostic or prognostic biomarkers. Their sensitivity to structural and compositional shifts within FF places these techniques as promising tools. They are capable of advancing reproductive bioanalysis and improving the characterization of follicular dysfunction.

## 3. Methodological Principles of FTIR and Raman Spectroscopy

To fully interpret the spectral fingerprints of endometriosis in FF, a foundational understanding of the analytical techniques is essential. This section highlights the core principles of FTIR and Raman spectroscopy, explaining the physical basis of how molecular vibrations are probed to generate a biochemical profile.

### 3.1. Fundamentals of FTIR Spectroscopy

#### 3.1.1. Theory

Fourier Transform Infrared (FTIR) spectroscopy is a significant technique. It probes the vibrational properties of molecules by measuring their absorption of infrared radiation [56,63]. FTIR relies on the principle that each chemical bond exhibits characteristic vibrational energies. These energies can be selectively excited when exposed to infrared light of matching frequency. When infrared radiation interacts with a sample, molecular bonds undergo stretching and bending transitions. This remains so provided that the vibration causes a measurable change in the dipole moment of the molecule, which is a prerequisite for Infrared (IR) activity [56].

In a typical FTIR instrument, the Michelson interferometer generates an interferogram. This is achieved by modulating the infrared beam through controlled path-length differences. This time-domain signal is then mathematically transformed into a frequency-domain spectrum using a Fourier transform algorithm [63]. Overall, this approach offers high spectral resolution. Other advantages include rapid acquisition and an improved signal-to-noise ratio compared to traditional dispersive infrared techniques. As such, this makes FTIR suitable for complex biological samples.

#### 3.1.2. Key Instrumentation and Measurement Modes

Modern FTIR spectrometers can operate in several modes. The Transmission and Attenuated Total Reflection (ATR) is the most common in biological analysis [57,63]. In transmission mode, the infrared beam passes directly through a thin sample layer. However, for aqueous systems such as biofluids, water’s strong IR absorption necessitates extremely short optical path lengths. These are often below 10 μm to avoid spectral saturation [56]. This requirement makes transmission measurements technically challenging, thus limiting their broader applicability in routine bioanalysis. The ATR mode has therefore become predominant. This is due to its minimal sample preparation, robustness, and suitability for heterogeneous or highly absorbing materials [55,57]. As a result, ATR is particularly advantageous for analyzing liquids, gels, and soft biological matrices, providing reproducible spectra even from minute sample volumes.

#### 3.1.3. Spectral Interpretation: Characteristic Bands for Biomolecules

The FTIR spectrum of follicular fluid (FF) reflects the complex and dynamic biochemical environment of the ovarian follicle, integrating systemic components derived from blood plasma with locally secreted molecules produced by granulosa and theca cells. As a clinical biofluid directly involved in oocyte maturation, FF shares certain compositional similarities with other reproductive biofluids and tissues, such as serum, endometrial tissue, and conditioned media from reproductive cell lines; however, its biochemical profile is uniquely shaped by follicular metabolism and endocrine regulation. Consequently, while the observed FTIR bands correspond to common biomolecular vibrations, their relative intensities, band shapes, and inter-band relationships provide FF-specific biochemical information.

Proteins represent the dominant molecular fraction of FF and are primarily responsible for the most intense infrared absorption features. The Amide I band, typically observed in the 1680–1620 cm^−1^ region and arising mainly from C=O stretching vibrations of the peptide backbone, is highly sensitive to protein secondary structure [55,56,57]. In the context of FF, variations in this band may reflect conformational changes in serum albumin, globulins, and locally produced signaling proteins, as well as interactions with lipids and metabolites. Similar Amide I profile alterations have been reported in FTIR studies of endometrial tissues and reproductive cell lines, where they were linked to changes in cellular differentiation and metabolic state [64,65]. The Amide II band, located around 1560–1520 cm^−1^ and associated with N–H bending and C–N stretching [55,56,57], further contributes to characterizing protein concentration and intermolecular interactions within FF, paralleling observations in other clinical biofluids such as serum and uterine fluid.

Lipid-related features constitute another important component of the FTIR spectrum of FF, reflecting the critical role of lipids in follicular development and oocyte competence. The strong C–H stretching vibrations of CH_2_ and CH_3_ groups in the 2800–3000 cm^−1^ region provide information on lipid abundance and hydrocarbon chain organization, while the ester carbonyl (C=O) stretching band near 1740 cm^−1^ is characteristic of phospholipids and triglycerides [57]. Comparable lipid-associated bands have been extensively described in FTIR analyses of endometrial tissues and cultured reproductive cell lines, where they were associated with membrane synthesis, steroidogenesis, lipids peroxidation [42,66]. In FF, these lipid features are particularly relevant, as they reflect both systemic lipid transport and local lipid remodeling essential for oocyte maturation [42,66].

The spectral region between 1200 and 900 cm^−1^, often referred to as the fingerprint region, encompasses overlapping contributions from carbohydrates, glycoproteins, and low-molecular-weight metabolites [55]. In FF, these bands are associated with glucose-derived compounds, glycosaminoglycans, and glycoproteins that contribute to the metabolic and structural support of the developing oocyte [67]. Similar spectral signatures have been reported in FTIR studies of uterine and endometrial fluids, where they were linked to changes in metabolic activity and cellular secretory function [68].

**Figure 3:**
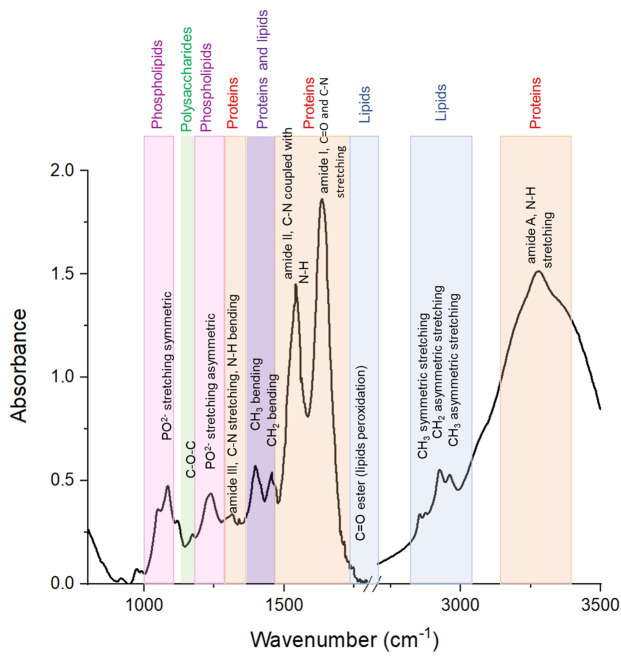
Representative schematic Raman spectrum of biological materials illustrating major vibrational bands commonly observed in biofluids and tissues. Highlighted regions correspond to proteins (Amide I, Amide II, and Amide III), lipids (C–H stretching and bending modes), carbohydrates, and phosphate-containing compounds. The figure is provided for illustrative purposes to facilitate spectral interpretation and does not represent experimental data from a specific biological sample.

Although nucleic acids are present in FF at relatively low concentrations, phosphate-containing compounds and extracellular nucleic acids released by follicular cells contribute to absorption bands around 1220–1240 cm^−1^, corresponding to asymmetric PO_2_^−^ stretching vibrations, as well as features in the 1000–1100 cm^−1^ region [57]. These signals are consistent with FTIR observations in endometrial tissues and reproductive cell cultures [66,69], where they have been associated with cellular turnover and extracellular vesicle activity. FTIR spectra of biological materials are presented in Figure 3.

#### 3.1.4. Advantages and Limitations

FTIR spectroscopy offers high sensitivity. As such, it is capable of detecting minute structural changes corresponding to bond length alterations smaller than 0.2 Å [56]. This sensitivity stems from the technique’s ability to resolve subtle variations in vibrational frequencies, which reflect even small perturbations in molecular geometry or chemical environment. FTIR also provides rapid data acquisition and is a well-established method for the analysis of complex biological fluids [55,57]. Its ease of use, compatibility with automated workflows, and broad availability have contributed to its widespread adoption in biomedical research.

Nevertheless, as advantageous as it is, FTIR spectroscopy also exhibits certain weaknesses. Its primary limitation is the strong absorption of water. Particularly, it is the H–O–H bending mode at ≈1645 cm^−1^, which overlaps with the crucial Amide I region. This interference can obscure protein-related spectral features. As a result, it requires careful sample handling, controlled drying, or the use of ATR to mitigate its effect [56,57]. Another challenge is the relatively low spatial resolution characteristic of the long-wavelength infrared radiation. Nevertheless, this can be partially improved by coupling FTIR with infrared microscopes [63]. Despite these limitations, FTIR remains a robust method for global biochemical profiling. This is more so when spectral preprocessing and multivariate analysis techniques are applied to enhance signal interpretability.

### 3.2. Fundamentals of Raman Spectroscopy

#### 3.2.1. Theory

Raman spectroscopy is based on the inelastic scattering of light, known as the Raman effect [60]. When monochromatic light interacts with a molecule, most photons undergo elastic Rayleigh scattering. However, a tiny fraction (≈1 in 10^7^ photons) is inelastically scattered. This produces a frequency shift that corresponds to the vibrational energy levels of the molecular bonds [70]. This shift, observed as Stokes or anti-Stokes scattering, encodes vibrational information that forms a unique molecular fingerprint. Unlike FTIR, Raman activity requires a change in molecular polarizability during vibration. This makes Raman spectroscopy especially sensitive to symmetric, non-polar bonds and complementary to infrared-based methods [58].

#### 3.2.2. Key Instrumentation

A standard Raman spectrometer consists of a laser excitation source, a spectrometer to disperse the scattered light, and a sensitive detector [62]. High-performance designs often incorporate notch or edge filters to suppress Rayleigh scattering. The result is an efficient collection of the weaker Raman signal. A major technological advancement is Confocal Raman Microscopy. This integrates a Raman spectrometer with a confocal optical microscope [70]. The system employs a spatial pinhole to reject out-of-focus light, resulting in excellent axial and lateral resolution. This configuration enables three-dimensional chemical mapping with sub-micron precision. In reproductive biology, this includes applications such as characterizing the biochemical composition of a single oocyte, cumulus cells, or spatial gradients within the follicular microenvironment [58,59].

#### 3.2.3. Spectral Interpretation: Characteristic Bands for Biomolecules

**Figure 4:**
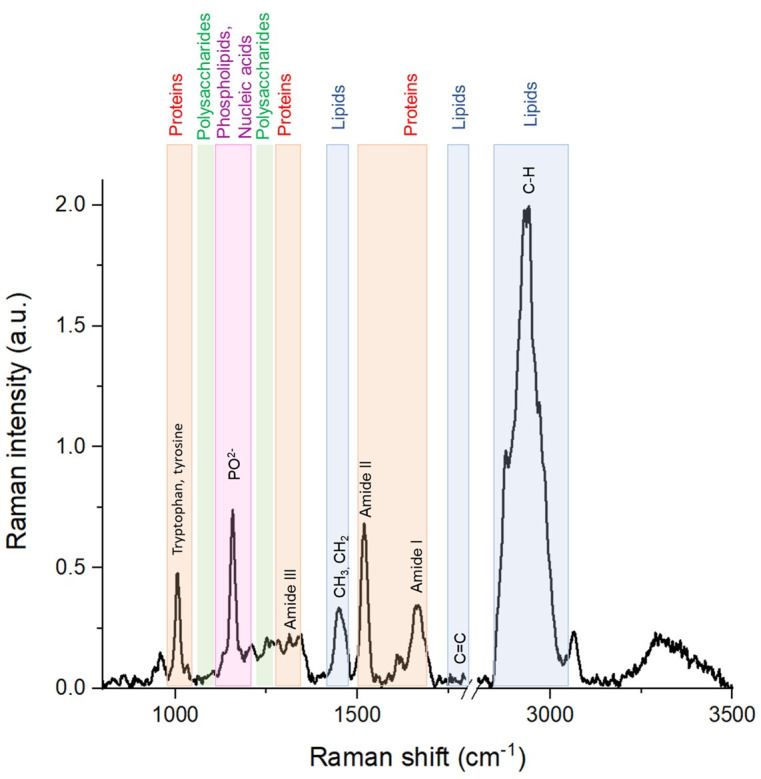
Representative schematic Raman spectrum of biological materials illustrating major vibrational bands commonly observed in biofluids and tissues. Highlighted regions correspond to proteins (Amide I, Amide II, and Amide III), lipids (C–H stretching and bending modes), carbohydrates, and phosphate-containing compounds. The figure is provided for illustrative purposes to facilitate spectral interpretation and does not represent experimental data from a specific biological sample.

Similar to observations in other reproductive biofluids, endometrial tissues, and reproductive cell lines, the Raman spectrum of FF is dominated by contributions from proteins and lipids, with additional signals arising from nucleic acids and metabolites, Figure 4.

Protein-related Raman features in FF are characterized by a relatively weak Amide I band in the 1650–1680 cm^−1^ region, while the Amide III band, appearing between 1230 and 1310 cm^−1^, provides more prominent information on protein backbone conformation. In addition, strong and well-defined Raman bands arising from aromatic amino acids, particularly phenylalanine at approximately 1003 cm^−1^, as well as tyrosine and tryptophan [61,62], serve as sensitive markers of protein composition and microenvironment. These aromatic amino acid signatures have been widely reported in Raman studies of serum, endometrial tissues, and cultured reproductive cells [71,72,73] where they were linked to protein expression levels and cellular functional state. In FF, such features reflect both systemic protein transport and local protein secretion within the follicle.

Lipid-associated Raman bands are among the most intense features observed in FF spectra. The C–H stretching region between 2800 and 3100 cm^−1^ provides detailed information on lipid content and molecular organization, while bands corresponding to C=C stretching vibrations around 1650–1680 cm^−1^ and CH_2_/CH_3_ bending modes near 1440 cm^−1^ offer insight into lipid unsaturation and acyl chain packing [58,59]. Similar Raman markers of lipid structure have been reported in studies of endometrial tissues and ovarian cell lines [73] where they were associated with membrane dynamics, steroid hormone synthesis, and metabolic activity. In the context of FF, these lipid features are particularly relevant, as lipid composition and degree of unsaturation have been linked to oocyte quality and developmental potential.

Raman signatures of nucleic acids, although less dominant, are detectable through characteristic vibrations of nucleotide bases in the 1200–1400 cm^−1^ and 1500–1800 cm^−1^ regions, as well as the symmetric stretching vibration of the phosphodiester backbone (PO_2_^−^) at approximately 1095 cm^−1^ [60,62]. These features are consistent with Raman observations in endometrial tissues and reproductive cell cultures [73] where they have been associated with cellular proliferation and extracellular nucleic acid release. In FF, they likely reflect follicular cell activity and the presence of extracellular vesicles or nucleic acid fragments.

Importantly, the diagnostic and analytical potential of Raman spectroscopy in FF does not rely on the identification of single, unique spectral bands. Instead, it lies in the analysis of multivariate spectral patterns that collectively describe the biochemical state of the follicular microenvironment.

#### 3.2.4. Advantages and Limitations

Vibrational spectroscopy techniques such as FTIR and Raman spectroscopy have been widely explored as label-free and minimally invasive tools in reproductive medicine; however, their strengths and limitations differ substantially depending on whether they are applied as in vitro assays or evaluated in vivo or ex vivo on animal or human tissues and biofluids. Distinguishing between these contexts is essential for assessing their translational and clinical relevance.

In in vitro settings, particularly in the analysis of biofluids such as follicular fluid or conditioned media from reproductive cell lines, both FTIR and Raman spectroscopy offer high analytical flexibility. Raman spectroscopy is especially advantageous for in vitro analysis of aqueous samples due to its weak sensitivity to water [59] enabling direct measurements of FF in its native liquid state without extensive sample preparation. Its high spatial resolution, reaching the sub-micron level when combined with confocal microscopy, allows localized biochemical analysis of individual cells, oocytes, or specific microdomains within a sample. These features make Raman spectroscopy well suited for exploratory and comparative in vitro studies aimed at identifying biochemical markers associated with oocyte quality or follicular health.

FTIR spectroscopy, while more sensitive to water absorption, is highly effective in in vitro applications when combined with attenuated total reflection (ATR) configurations or controlled drying protocols. In such settings, FTIR provides robust and reproducible biochemical fingerprints that reflect global molecular composition, making it particularly suitable for high-throughput screening and comparative analysis of multiple FF samples. However, in vitro measurements for both techniques may be influenced by sample handling, dehydration-induced conformational changes, and substrate effects, which limit direct extrapolation to physiological conditions if not carefully controlled.

In contrast, in vivo applications of vibrational spectroscopy face significantly greater technical and biological challenges. Direct in vivo FTIR measurements are generally impractical due to strong water absorption, limited penetration depth, and difficulties in optical access. Raman spectroscopy has shown greater potential for in vivo or minimally invasive applications, particularly through the development of fiber-optic probes and endoscopic Raman systems that enable measurements on animal or human tissues [74]. These approaches have been explored in various biomedical contexts, including reproductive tissues, where Raman spectroscopy has been used ex vivo or in situ to assess tissue composition. Nevertheless, in vivo Raman measurements are constrained by weak signal intensity, motion artifacts, tissue heterogeneity, and safety considerations related to laser exposure.

Ex vivo studies on animal or human tissues represent an intermediate translational stage between in vitro experiments and in vivo applications. Both FTIR and Raman spectroscopy have been successfully applied to ex vivo reproductive tissues, such as ovarian or endometrial samples, where they provide spatially resolved biochemical information while maintaining structural integrity [75]. These studies offer valuable insights into tissue-level biochemical organization but are inherently limited by post-excision changes and do not fully capture dynamic physiological processes.

Overall, while FTIR and Raman spectroscopy are highly effective as nondestructive in vitro analytical tools for studying reproductive biofluids and cells, their current clinical applicability is more advanced for ex vivo analysis than for routine in vivo diagnostics. Continued technological developments, including improved probe design, signal enhancement strategies, and standardized protocols, are required to bridge the gap between in vitro research applications and clinically relevant in vivo use.

#### 3.2.5. Measurement Conditions and Sample Preparation for FTIR and Raman Analysis of Follicular Fluid

Although FTIR and Raman spectroscopy offer significant advantages as label-free and non-destructive analytical techniques, their application to biological specimens such as follicular fluid (FF) is strongly influenced by measurement conditions, substrate selection, and sample preparation. Given the complex and highly aqueous nature of FF, careful optimization of these parameters is essential to ensure reproducibility, spectral quality, and biological relevance.

One of the primary considerations in spectroscopic analysis of FF is the physical state of the sample during measurement. Measurements can be performed either in the native liquid state or after controlled drying. Liquid-phase measurements preserve the physiological environment of FF and minimize structural alterations of biomolecules; however, in FTIR spectroscopy, they are challenged by the strong infrared absorption of water, particularly in the O–H stretching (~3400 cm^−1^) and bending (~1640 cm^−1^) regions, which can obscure protein-related bands [76]. To mitigate this effect, attenuated total reflection (ATR)-FTIR is commonly employed, as it reduces the effective optical path length and enables direct analysis of small volumes of aqueous biofluids [77]. In contrast, Raman spectroscopy is inherently less sensitive to water, making it well suited for measurements of FF in its native liquid form.

Alternatively, dried or partially dehydrated FF samples are frequently used to enhance spectral contrast and improve signal-to-noise ratios in both FTIR and Raman measurements. Drying concentrates biomolecular components and reduces spectral interference from water, allowing clearer observation of protein, lipid, and metabolite bands. However, dehydration may induce conformational changes in proteins or alter lipid organization, which should be considered when interpreting spectral features [78,79]. For this reason, drying protocols should be standardized, including temperature, drying time, and ambient conditions, to ensure comparability between samples.

Substrate selection represents another critical factor influencing spectral quality. For FTIR measurements, ATR crystals such as diamond, zinc selenide (ZnSe), or germanium are widely used due to their chemical inertness and compatibility with aqueous samples. Transmission FTIR measurements may employ calcium fluoride (CaF_2_) or barium fluoride (BaF_2_) windows when thin liquid films or dried residues are analyzed [80]. In Raman spectroscopy, low-background substrates such as CaF_2_, quartz, or polished silicon are preferred to minimize substrate-related spectral contributions [81]. For enhanced sensitivity, surface-enhanced Raman spectroscopy (SERS) substrates based on noble metal nanostructures may be used, although their application introduces additional variability related to surface chemistry and analyte adsorption [82].

Measurement parameters, including laser wavelength and power (for Raman), spectral resolution, acquisition time, and number of accumulations, must also be carefully optimized to avoid sample degradation and thermal effects. Low laser power and short acquisition times are recommended to prevent localized heating, particularly in dried samples.

Finally, pre-analytical factors such as sample handling, storage conditions, and freeze–thaw cycles can significantly influence the biochemical composition of FF and, consequently, its spectral profile. To preserve sample integrity, FF should be stored at low temperatures and subjected to minimal freeze–thaw cycles prior to spectroscopic analysis. Consistent protocols for centrifugation, filtration, and dilution should be applied to reduce variability introduced by cellular debris or protein aggregation.

By explicitly addressing measurement conditions, substrate choices, and sample state, the application of FTIR and Raman spectroscopy to follicular fluid can be standardized and optimized. These methodological considerations are essential for translating these techniques into reliable tools for comparative studies and future clinical or research applications involving FF.

#### 3.2.6. Practical Considerations for Clinical Implementation

Translating FTIR and Raman spectroscopy from research settings to routine IVF laboratory practice requires careful consideration of several practical parameters that directly impact clinical utility.

Turnaround time and workflow integration: Current spectroscopic measurements can be completed within 5–15 min per sample, including spectral acquisition and basic quality assessment. However, comprehensive data analysis involving spectral preprocessing and multivariate statistical modeling typically requires additional time (30–60 min) when performed by trained personnel. For routine clinical application, development of automated analysis pipelines with pre-validated classification algorithms could reduce total turnaround time to under 30 min, making this compatible with standard IVF workflow timelines where oocyte retrieval and insemination occur within 2–4 h.

Cost-effectiveness analysis: The primary equipment costs for a research-grade FTIR spectrometer range from €25,000–€50,000, while confocal Raman systems are more expensive (€50,000–€150,000). However, per-sample consumable costs are minimal (€1–5), primarily limited to disposable sample holders or ATR crystal cleaning materials. This compares favorably with targeted biochemical assays (ELISA: €10–30 per analyte) and omics-based approaches (proteomics/metabolomics: €100–300 per sample). The ability to obtain comprehensive biochemical profiles from a single measurement without additional reagents suggests potential for cost savings in research contexts, though clinical adoption would require demonstration of improved outcomes justifying equipment investment.

Standardization challenges: Several pre-analytical and analytical variables require rigorous standardization before clinical deployment:

• Follicular fluid collection: needle type, aspiration pressure, and avoidance of blood contamination;
• Sample processing: centrifugation parameters, aliquot volume, storage temperature, and freeze–thaw cycles;
• Measurement conditions: temperature, humidity, and calibration frequency;
• Spectral acquisition: laser power (Raman), number of scans, resolution, and background correction;
• Data analysis: preprocessing algorithms, baseline correction methods, and model updating protocols.

International consensus guidelines, similar to those developed for clinical NMR metabolomics, would be necessary to ensure inter-laboratory reproducibility and enable multi-center validation studies.

### 3.3. Complementary Nature of FTIR and Raman

The synergistic use of FTIR and Raman spectroscopy provides a far more comprehensive biochemical profile of a complex sample like follicular fluid than either technique could alone. This complementarity stems from their fundamental difference in physical mechanism: FTIR measures the absorption of infrared light due to a change in a molecule’s dipole moment. On the other hand, Raman measures the inelastic scattering of light due to a change in a molecule’s polarizability [58,60].

This fundamental distinction makes each technique uniquely sensitive to different molecular vibrations. For instance, as summarized in Table 1 below, FTIR is highly sensitive to polar bonds such as the C=O and C–O stretches. This makes it exceptionally strong for characterizing carbonyl groups in proteins (Amide I) and lipids (ester C=O) [56,57]. In contrast, Raman spectroscopy excels at probing symmetric, non-polar bonds and ring structures. Key examples are the C=C stretches in unsaturated lipids and aromatic rings in amino acids (Phe, Tyr) and nucleic acid bases, which often yield weak or silent signals in FTIR [61,62]. Furthermore, their differing interaction with water—a strong absorber in FTIR but a weak scatterer in Raman—means that Raman is ideal for hydrated samples, while FTIR requires careful handling to mitigate water interference [56,59]. By integrating data from both techniques, one can achieve a more complete and cross-validated assessment of the full spectrum of biomolecules (proteins, lipids, nucleic acids, and carbohydrates) within the follicular microenvironment.

While exact limits of detection (LOD) and limits of quantification (LOQ) are largely instrument- and configuration-dependent, studies indicate that Raman spectroscopy can detect biomolecules in follicular fluid at micromolar to low millimolar concentrations, while FTIR demonstrates comparable sensitivity but is more affected by sample hydration and path length [83]. These limitations, including susceptibility to fluorescence interference (Raman) and strong water absorption (FTIR), should be considered when designing experiments and interpreting spectral data. By integrating data from both techniques, researchers can achieve a more complete and cross-validated assessment of the full spectrum of biomolecules in FF, enhancing the identification of potential biochemical markers relevant to oocyte quality and reproductive health.

Importantly, when interpreting spectroscopic data from follicular fluid, it must be recognized that the molecular signatures detected represent the complex extracellular environment that bathes the cumulus–oocyte complex, rather than the intracellular composition of the oocyte itself. However, because cumulus cells actively transport molecules between FF and the oocyte via gap junctions, and because oocyte-secreted factors influence cumulus cell gene expression and metabolism, FF composition is intrinsically linked to oocyte health [42,52]. Spectroscopic analysis of FF thus provides an integrated readout of the metabolic and regulatory state of the entire follicular unit, including contributions from the oocyte, cumulus cells, and granulosa cells. This makes it a powerful surrogate to assess the conditions that determine oocyte developmental competence.

## 4. The Untapped Potential: Spectroscopic Analysis of FF in Reproductive Disorders

The pathophysiological mechanisms linking endometriosis to infertility are well recognized. However, a critical translational gap remains. This is the lack of a robust, direct method to assess the functional health of the oocyte and its microenvironment. As established, FF serves as a rich reservoir of biochemical information directly reflecting this status. However, the application of FTIR and Raman spectroscopy to this specific biofluid, mainly in the context of endometriosis, remains largely unexplored. This section will synthesize evidence from related fields to illustrate the considerable potential of these methods.

### 4.1. The Established Link Between FF Composition and Oocyte Health

Literature from various ‘omics’ disciplines confirms that the biochemical composition of FF is profoundly altered in infertility disorders. This establishes it as a critical determinant of oocyte quality and a rich source of meaningful biomarkers. In endometriosis, proteomic analyses have consistently identified a distinct FF protein profile. It is often characterized by dysregulation of proteins involved in immune response, oxidative stress response, and vitamin D metabolism. These collectively contribute to a compromised follicular environment and poorer IVF outcomes [84,85]. This proteomic dysregulation is coupled with significant metabolomic agitations. Recent metabolomic studies reveal alterations in amino acid metabolism within the FF of endometriosis patients, including elevated levels of branched-chain amino acids (BCAAs) and glutamate. This is indicative of underlying oxidative stress and mitochondrial dysfunction that can impair oocyte maturation [86,87]. Further, specific metabolic shifts, such as changes in lipid and energy pathways, have been directly linked to the reduced fertilization potential of oocytes from women with the disease [88,89].

This phenomenon of a disease-specific FF signature extends beyond endometriosis. In Polycystic Ovary Syndrome (PCOS), proteomic studies reveal significant alterations in the FF proteome. This includes aberrant expression of proteins involved in coagulation, inflammation, and, critically, mitochondrial oxidative phosphorylation. That may explain the impaired oocyte competence observed despite high antral follicle counts [49,90]. Metabolomic profiling further supports this. It shows unique FF metabolic fingerprints across various infertility etiologies, including diminished ovarian reserve (DOR). This can differentiate between patient groups and even show predictive value for clinical outcomes like embryo quality and implantation [87,91].

In summary, research across proteomics and metabolomics has significantly demonstrated that the FF microenvironment harbors a specific biochemical signature reflective of systemic and ovarian pathology. These ‘omics’ studies validate that the pathophysiological state of endometriosis and other infertility conditions is accurately imprinted on the FF composition. This confirms that a detectable and meaningful biochemical signature exists and is directly relevant to oocyte health and reproductive success.

### 4.2. Spectroscopic and Metabolomic Alterations in Follicular Fluid of Women with Endometriosis

Recent advances in metabolomics and spectroscopic techniques, particularly proton nuclear magnetic resonance (^1^H-NMR) spectroscopy and liquid chromatography–mass spectrometry (LC-MS), have enabled detailed characterization of follicular fluid (FF) composition in women with endometriosis. These studies demonstrate significant alterations in metabolic pathways related to energy metabolism, oxidative stress, amino acid turnover, and lipid metabolism. Several investigations using ^1^H-NMR metabolomics have reported increased concentrations of lactate and pyruvate in FF from women with endometriosis, suggesting a shift toward enhanced glycolytic activity and altered mitochondrial metabolism. Concurrently, decreased glucose levels have been observed, supporting the hypothesis of dysregulated energy metabolism within the follicular microenvironment. Alterations in amino acid profiles have also been documented. Changes in alanine, tyrosine, leucine, proline, and glycine concentrations indicate disturbances in amino acid metabolism that may affect oocyte competence and embryo development. Additionally, lipidomic analyses revealed modifications in lipid species and free fatty acids, which may reflect inflammatory processes and oxidative imbalance characteristic of endometriosis. Furthermore, studies employing LC-MS have identified increased oxidative stress markers and disturbances in steroid-related metabolites in FF samples from affected patients. Collectively, these findings support the concept that endometriosis significantly modifies the biochemical milieu of the ovarian follicle, potentially contributing to impaired fertility. A summary of selected studies is presented in Table 2.

### 4.3. Vibrational Spectroscopy in Reproductive Medicine

The direct application of vibrational spectroscopy to endometriosis-affected FF is limited. However, its successful deployment across other reproductive tissues and biofluids powerfully demonstrates its sensitivity and diagnostic potential in gynecological and reproductive contexts. Most importantly, studies have already confirmed that FTIR and Raman spectroscopy can detect a distinct spectral signature of endometriosis itself. For instance, cervical swab analysis using FTIR demonstrated a sensitivity of approximately 87–90% and specificity of 85–88% for differentiating women with endometriosis from healthy controls [96,97]. That highlights its potential as a non-invasive diagnostic tool. Furthermore, investigations at the cellular level have proven that these techniques are exquisitely sensitive to the disease’s local microenvironment. In particular, Raman and FTIR analysis of granulosa cells from patients with unilateral ovarian endometriosis revealed biochemical alterations in cells from the contralateral, unaffected ovary, achieving classification accuracies of 82–85% [72,98], thus demonstrating a systemic impact of the disease that is detectable by both FTIR and Raman microspectroscopy.

Beyond endometriosis detection, vibrational spectroscopy has shown great promise in assessing functional reproductive potential. A pioneering FTIR study quantified differences between follicular fluids from large versus small antral follicles, reporting clear spectral distinctions corresponding to variations in protein and lipid content, with classification accuracy exceeding 80% [99]. More recently, Raman spectroscopy has been successfully applied to FF for oocyte quality assessment in Polycystic Ovary Syndrome (PCOS). Specific spectral patterns identified by Raman spectroscopy allowed differentiation between mature and immature oocytes with sensitivity and specificity values around 78–83% [100]. This approach has been extended to screening applications; Raman analysis of FF and plasma combined with machine learning algorithms achieved an overall classification accuracy of 85–88% in identifying women with PCOS [101]. It can also be applied to cases of unexplained infertility, where markers of oxidative stress detected by Raman spectroscopy combined with machine learning distinguished affected individuals with an accuracy of 80–84% [102].

The utility of these techniques extends to other gynecological pathologies. For example, simultaneous FTIR and Raman spectroscopy distinguished endometrial hyperplasia from endometrial cancer with reported classification accuracies of 86–89% [103]. Overall, this existing body of work strongly validates the argument that these spectroscopic methods are highly promising for uncovering the specific biochemical disruption that endometriosis causes in the FF microenvironment. A summary of vibrational spectroscopy studies in reproductive and gynecological contexts is presented in Table 3.

### 4.4. The Invasiveness Limitation: Follicular Fluid Collection Requires Oocyte Retrieval

A fundamental limitation of follicular fluid-based diagnostics that must be acknowledged is the invasive nature of sample collection. Follicular fluid can only be obtained through transvaginal ultrasound-guided follicular aspiration, a procedure that is performed as part of oocyte retrieval for in vitro fertilization (IVF) or intracytoplasmic sperm injection (ICSI) cycles. This procedure requires:

• Controlled ovarian stimulation with gonadotropins (typically 10–14 days);
• Transvaginal ultrasound-guided needle aspiration under sedation or general anesthesia;
• Specialized clinical expertise and operating room facilities;
• Significant financial cost (typically €1000–3000 for the retrieval procedure alone).

Consequently, follicular fluid analysis is inherently restricted to women undergoing assisted reproductive technology (ART). This represents a substantial limitation because:

1. Not all women with endometriosis pursue IVF: Many women with endometriosis-associated infertility attempt natural conception or undergo less invasive treatments such as intrauterine insemination (IUI) before progressing to IVF. Estimates suggest that only 30–50% of women with endometriosis-related infertility ultimately undergo IVF [8,9]. For the remaining women, follicular fluid is not accessible for diagnostic purposes.
2. Diagnostic applications are precluded: If FTIR/Raman analysis of follicular fluid were developed as a diagnostic tool for endometriosis or a prognostic marker for fertility potential, it could only be offered to women already committed to IVF. This would exclude women seeking:
  • Initial diagnostic evaluation for suspected endometriosis;
  • Fertility assessment before attempting natural conception;
  • Monitoring of disease progression or treatment response;
  • Evaluation of women with unexplained infertility who may have occult endometriosis [7].
3. Research applications are limited to ART populations: Studies using follicular fluid are necessarily restricted to women undergoing IVF, introducing potential selection bias. Women with endometriosis who conceive naturally or who do not pursue ART may have different disease characteristics, severity, or fertility potential than those who require IVF. Findings from follicular fluid studies may not generalize to the broader endometriosis population.
4. Longitudinal sampling is impractical: Follicular fluid can only be collected at the time of oocyte retrieval, providing a single snapshot. Repeated sampling to track disease progression, response to medical therapy, or changes over time is not feasible.

### 4.5. Alternative Biofluids for Less Invasive Spectroscopic Analysis

Given the invasiveness of follicular fluid collection, it is important to consider whether other, more accessible biofluids might serve as suitable alternatives for spectroscopic analysis in women with endometriosis, particularly those not undergoing IVF. Table 4 summarizes the potential advantages and limitations of alternative biofluids.

#### Cervical Mucus and Swabs: Established FTIR Applications

As discussed in Section 4.3, cervical swab analysis using FTIR has demonstrated encouraging diagnostic performance for endometriosis, with reported sensitivity of 87–90% and specificity of 85–88% [96,97]. Cervical sampling is minimally invasive, inexpensive, and easily performed in any gynecological clinic. The diagnostic signal presumably derives from endometrial cells shed into the cervical canal, which may carry biochemical signatures of the disease. Cervical mucus, which can be collected by simple aspiration, represents another accessible biofluid that reflects the hormonal and inflammatory state of the upper genital tract. Its viscoelastic properties and biochemical composition change throughout the menstrual cycle, but standardized collection protocols could enable meaningful comparisons.

### 4.6. The Critical Knowledge Gap

Having acknowledged the invasiveness limitation of follicular fluid collection and explored alternative biofluids that may offer less invasive options for spectroscopic analysis, we now return to the central knowledge gap that motivates this review. Despite the proven utility of FTIR and Raman spectroscopy in related applications, and despite the wealth of information contained within follicular fluid, direct application of these techniques to endometriosis-affected follicular fluid remains unexplored. However, it is essential to recognize that the proposed research framework below is specifically relevant to women undergoing IVF, and findings may not generalize to the broader endometriosis population. Complementary studies using more accessible biofluids will be necessary to develop diagnostic tools applicable to all women with endometriosis.

The evidence synthesized herein presents a compelling yet unresolved scientific opportunity. On one hand, the FF is well established as a critical microenvironment. Its biochemical composition is significantly altered in endometriosis and directly linked to compromised oocyte quality [84,86]. On the other hand, FTIR and Raman spectroscopy have been rigorously validated as powerful, sensitive, and label-free techniques. They are capable of detecting the spectral signature of endometriosis in cervical swabs and granulosa cells [72,96]. They can also assess oocyte developmental competence from FF in other conditions like PCOS [100].

Despite this powerful combination of rationale and methodology, a striking and critical knowledge gap persists. There is a significant lack of comprehensive, dedicated studies applying FTIR and Raman spectroscopy specifically to the analysis of FF from women with endometriosis. The techniques have proven their merit in adjacent applications. Alternatively, the biofluid has been shown to be information–rich. However, the direct interrogation of the endometriosis-affected follicular environment using these holistic spectroscopic tools remains largely unexplored. This gap represents a significant missed opportunity to obtain a complete, untargeted biochemical fingerprint of the disease’s impact on the oocyte’s immediate surroundings. Therefore, the central element of this review is to highlight this specific void. Bridging it is the logical and essential next step for advancing our understanding of endometriosis -associated infertility. The application of FTIR and Raman spectroscopy to this specific context is not merely an incremental addition. Rather, it is fundamental to uncover novel, integrated pathophysiological insights and biomarker profiles that have thus far remained elusive.

### 4.7. Rationale and Proposed Framework for Future Research

The narrative constructed throughout this review arrives at a powerful and unmet scientific opportunity. Endometriosis is a prevalent and debilitating cause of infertility. Its pathophysiology impairs oocyte quality through mechanisms that are yet to be fully grasped. The FF, which is the immediate microenvironment of the developing oocyte, has been well established by proteomics and metabolomics as a rich source of biochemical information that reflects this pathology [85,86]. Concurrently, FTIR and Raman spectroscopy have emerged as powerful, complementary analytical techniques. They are uniquely suited for the holistic analysis of such complex biofluids. A case in point is their proven efficacy in detecting endometriosis in other sample types and evaluating oocyte competence in related conditions like PCOS [72,96,100]. This highlights the potential of the methods in clinical diagnosis. Nevertheless, the absence of dedicated studies applying these spectroscopic techniques specifically to the FF of women with endometriosis persists.

To translate this conceptual opportunity into an operational research pathway, we propose a pilot prospective case–control study designed to evaluate the diagnostic and predictive utility of FTIR and Raman spectroscopy applied to FF samples. However, before outlining the proposed research framework, it is important to explicitly state the scope and limitations of this approach. The following study design is specifically intended for the IVF population and addresses the question of whether follicular fluid spectroscopy can provide clinically useful information about oocyte quality and endometriosis-associated infertility in women already undergoing ART. This research would not yield a diagnostic tool applicable to all women with endometriosis, nor would it replace less invasive diagnostic approaches. Rather, it aims to:

• Elucidate the biochemical impact of endometriosis on the follicular microenvironment;
• Identify spectral signatures associated with oocyte developmental competence in endometriosis patients;
• Potentially develop a prognostic tool to guide clinical decisions during IVF cycles such as insemination method, embryo culture strategies, or cryopreservation decisions.

*(a)*  *Proposed Study Design:* A prospective case–control study would be conducted in women undergoing IVF treatment. The study group would include women with laparoscopically confirmed endometriosis, while the control group would consist of women undergoing IVF due to male-factor infertility, with no clinical or surgical evidence of endometriosis.
*(b)*  *Inclusion criteria:* Age 20–38 years, BMI 18–30 and standardized ovarian stimulation protocols. Exclusion criteria would include PCOS, diminished ovarian reserve, autoimmune disorders, metabolic disease, or prior ovarian surgery unrelated to endometriosis.
*(c)*  *Sample Size Considerations:* As an exploratory pilot study aiming to identify spectroscopic signatures and estimate effect sizes, an initial cohort of approximately 25–30 patients per group would be appropriate. This sample size is consistent with prior metabolomic feasibility studies and would allow preliminary multivariate modeling while minimizing overfitting. Power calculations for a subsequent validation study would be based on effect sizes derived from this pilot dataset.
*(d)*  *Standardization of FF Collection and Processing*: To ensure reproducibility and minimize pre-analytical variability, FF should be collected from dominant follicles (>18 mm) during oocyte retrieval, avoiding visible blood contamination. Samples should be centrifuged immediately (e.g., 3000× *g* for 10 min), aliquoted, and stored at −80 °C until analysis. Freeze–thaw cycles must be avoided. Detailed recording of stimulation protocol, hormone levels, and follicular size should accompany each sample.
*(e)*  *Experimental Workflow: The proposed analytical pipeline would include*: sample preparation under standardized conditions (thawing on ice, homogenization), acquisition of FTIR and Raman spectra using calibrated instrumentation with controlled acquisition parameters, spectral preprocessing (baseline correction, normalization, smoothing, cosmic ray removal for Raman), and multivariate statistical analysis.
*(f)*  *Primary and Secondary Endpoints:* Primary endpoint: identification of a spectroscopic signature distinguishing FF from endometriosis patients and controls. Secondary endpoints: correlation between spectroscopic profiles and oocyte maturity (MII rate).
*(g)*  *Statistical Modeling Strategy:* Unsupervised methods such as Principal Component Analysis (PCA) would first be applied to detect clustering patterns and outliers. Supervised models (e.g., Partial Least Squares–Discriminant Analysis, Support Vector Machines, or Random Forest classifiers) would then be used to construct predictive models. Internal validation would be performed using cross-validation (e.g., k-fold or leave-one-out), and model performance would be evaluated through accuracy, sensitivity, specificity, and area under the ROC curve (AUC). Feature importance analysis would identify the most discriminative spectral regions corresponding to biochemical alterations (lipids, proteins, nucleic acids). Where feasible, integration with metabolomic datasets could enable multimodal modeling approaches to strengthen biological interpretation.
*(h)*  *Translational Roadmap and Clinical Implementation Pathway.* Beyond the pilot case–control study proposed above, a structured translational pathway is necessary to bridge the gap between proof-of-concept research and routine clinical application:
  • Phase 1: Technical validation (current stage)—Establish reproducible measurement protocols, define spectral acquisition parameters, and develop preliminary classification models in well-characterized cohorts.
  • Phase 2: Multi-center clinical validation—Conduct prospective studies across multiple IVF centers to:
    ○ Validate diagnostic accuracy in diverse patient populations;
    ○ Assess inter-laboratory reproducibility using standardized protocols;
    ○ Define reference ranges and quality control metrics;
    ○ Establish normative spectral databases for different infertility etiologies.
  • Phase 3: Health technology assessment—Evaluate:
    ○ Incremental clinical utility beyond existing biomarkers (AMH, AFC, hormone levels);
    ○ Impact on clinical decision-making and IVF outcomes;
    ○ Cost-effectiveness analysis including equipment, training, and quality assurance;
    ○ Patient and provider acceptability.
  • Phase 4: Regulatory approval and commercialization—Depending on intended use:
    ○ For research-use-only (RUO) applications: laboratory-developed test validation;
    ○ For in vitro diagnostic (IVD) use: regulatory pathways (CE marking under IVDR in Europe, FDA clearance in US) requiring analytical and clinical performance studies;
    ○ Development of user-friendly software interfaces with automated spectral interpretation;
    ○ Training programs for clinical laboratory personnel.
  • Comparison with existing biomarkers: Current clinical assessment of oocyte quality and IVF prognosis relies on:
    ○ Hormonal markers: AMH, FSH, estradiol (reflect ovarian reserve but not direct oocyte competence);
    ○ Ultrasound parameters: AFC, follicular size (anatomical rather than functional);
    ○ Dynamic tests: Ovarian stimulation response (retrospective rather than predictive);
    ○ Embryo morphology: Assessed after fertilization (cannot guide insemination decisions).

FTIR/Raman spectroscopy of follicular fluid offers potential advantages:

• Direct assessment of the oocyte’s microenvironment;
• Holistic biochemical profiling rather than single analytes;
• Results available before insemination, potentially guiding clinical decisions;
• Non-destructive analysis requiring minimal sample volume (50–100 μL).

However, prospective studies demonstrating superior or complementary predictive value compared to existing biomarkers are essential before clinical adoption can be recommended.

## 5. Conclusions

Endometriosis remains a major cause of infertility. Its detrimental impact on oocyte quality and embryonic competence represent a critical yet inadequately understood clinical challenge. This review has synthesized evidence establishing that FF serves as a vital window into this compromised ovarian microenvironment. Specifically, its altered composition reflects the underlying pathology. Despite traditional ‘omics’ having begun to catalog these changes, the holistic, analytical power of FTIR and Raman spectroscopy offer a uniquely promising avenue to decode the full biochemical fingerprint of the disease. The two have proven utility in diagnosing endometriosis in other tissues and assessing oocyte health in related infertility conditions. However, their targeted application to the FF of endometriosis patients presents a significant and untapped frontier.

Hence, from this identified gap, targeted research studies are needed. By leveraging the synergistic strengths of FTIR and Raman spectroscopy, this investigative framework can uncover the distinct spectral signatures that define endometriosis within the follicular niche. Success in this endeavor holds the potential to revolutionize our understanding. This is specifically by providing significant molecular-level insight into the mechanisms of oocyte compromise, moving beyond correlation to mechanism.

However, realizing this potential requires more than successful research applications. A structured translational pathway must address practical considerations. This includes turnaround time compatible with IVF workflows, cost-effectiveness relative to existing diagnostic approaches, rigorous standardization protocols to ensure inter-laboratory reproducibility, and regulatory validation for clinical use. The proposed multi-phase framework entails progressing from technical validation through multi-center clinical studies to health technology assessment. It provides a roadmap for translating spectroscopic follicular fluid analysis from bench to bedside. If successfully navigated, this approach could establish vibrational spectroscopy as a valuable adjunct to existing biomarkers. As such, it will offer clinically actionable information about oocyte quality before insemination and potentially improve patient counseling, treatment personalization, and ultimately, reproductive outcomes for women with endometriosis-associated infertility.

## Figures and Tables

**Table 1:** Comparative Summary of FTIR vs. Raman Spectroscopy for Follicular Fluid Analysis.

Feature	FTIR Spectroscopy	Raman Spectroscopy
Physical Principle	Absorption of IR light; requires a change in dipole moment.	Inelastic scattering of light; requires a change in polarizability.
Sensitivity to Water	Strong absorption, especially ~1645 cm^−1^ (H–O–H bend); requires minimal water or ATR mode.	Minimal interference; water bands are weak, ideal for aqueous samples.
Spatial Resolution	Limited (typically >10–20 µm); can be improved with microscopy.	Excellent (<1 µm with confocal microscopy); suitable for single-cell/oocyte analysis.
Key Biomolecule Strengths	− Proteins: Strong Amide I & II bands. − Lipids: Strong C=O ester stretch. − Carbohydrates: Fingerprint region.	− Proteins: Strong Amide III, aromatic amino acids (Phe ~1003 cm^−1^). − Lipids: C=C stretches, C-H bends. − Nucleic Acids: Strong nucleotide base & backbone bands.
Signal Strength	Inherently strong signals.	Inherently weak signal (requires sensitive detectors).
Major Limitation	Strong water absorption obscures key regions.	Fluorescence interference can overwhelm the Raman signal.
Sample Preparation	Can require drying or thin films for transmission; ATR simplifies preparation.	Minimal; can analyze samples directly in aqueous media.
Acquisition Time	Typically fast (seconds to minutes).	Can be longer (minutes to hours) due to weak signal.

**Table 2:** Alterations in Follicular Fluid Composition in Women with Endometriosis.

References	Method of Spectroscopy/Analysis	Molecules Increased in Endometriosis	Molecules Decreased in Endometriosis
Marianna et al. [92]	^1^H-NMR metabolomics	Lactate, Pyruvate, Valine	–
Yu et al. [93]	NMR metabolomics	Total lipids, inflammatory-related metabolites	Leucine, Lysine, Proline, Free fatty acids
Guo et al. [94]	LC-MS metabolomics	Proline, Arginine, Threonine, Glycine	Selected steroid-related metabolites
Li et al. [95]	LC-MS metabolomics	Oxidative stress–related metabolites (e.g., chlorinated tyrosine derivatives)	Steroidogenesis-related metabolites

**Table 3:** Comparative Summary of vibrational spectroscopy studies in reproductive and gynecological contexts.

Ref.	Pathology/Context	Sample Type	Spectroscopy Method	Analytical Model	N (If Reported)	Performance Metrics	Validation Strategy	Key Limitations
[96] Karakaşlı et al., 2025	Endometriosis (endometrioma)	Cervical swabs	FTIR	Not specified	Not specified	Sensitivity ~87–90%; Specificity ~85–88%	Not specified	Limited detail on external validation; potential cohort size limitations
[97] Bozdag et al., 2019	Endometriosis	Cervical swabs	FTIR	Not specified	Not specified	Sensitivity ~87–90%; Specificity ~85–88%	Not specified	Likely internal validation; limited information on reproducibility
[98] Gioacchini et al., 2015	Unilateral ovarian endometriosis	Granulosa cells	FTIR (microspectroscopy)	Not specified	Not specified	Classification accuracy 82–85%	Not specified	Cellular-level study; limited direct clinical translation
[99] Thomas et al., 2000	Follicular development	Follicular fluid (large vs. small follicles)	FTIR	Classification model (unspecified)	Not specified	Accuracy > 80%	Not specified	Early study; potential small cohort; no external validation reported
[100] Huang et al., 2021	PCOS–oocyte maturity	Follicular fluid	Raman	Pattern recognition	Not specified	Sensitivity & Specificity ~78–83%	Not specified	Focused on oocyte competence; unclear external validation
[101] Zhang et al., 2021	PCOS screening	Follicular fluid & plasma	Raman + ML	Machine learning algorithms	Not specified	Accuracy 85–88%	ML-based (type not specified)	Risk of overfitting if limited cohort; cross-platform reproducibility not discussed
[102] Depciuch et al., 2023	Unexplained infertility	Follicular fluid & serum	Raman + ML	Machine learning	Not specified	Accuracy 80–84%	ML-based (type not specified)	Oxidative stress markers; generalizability requires external validation
[103] Barnas et al., 2019	Endometrial hyperplasia vs. cancer	Tissue samples	FTIR + Raman	Classification model	Not specified	Accuracy 86–89%	Not specified	Tissue-based study; applicability to non-invasive diagnostics limited

**Table 4:** Comparison of Biofluids for Spectroscopic Analysis in Endometriosis.

Biofluid	Collection Method	Invasiveness	Relevance to Endometriosis	Suitability for Non-IVF Patients	Key Limitations
Follicular fluid	Transvaginal aspiration (oocyte retrieval)	High (sedation required)	Direct oocyte microenvironment; reflects ovarian and follicular health	Only during IVF	Not accessible for natural conception; single timepoint
Uterine fluid	Uterine lavage or aspiration (catheter)	Moderate (outpatient, no sedation)	Reflects endometrial receptivity; may contain endometrial secretions	Yes	Cyclic variation; small volumes; limited proteomic characterization
Tubal fluid	Laparoscopic aspiration or transcervical balloon catheter	Moderate-high	Directly from implantation site; reflects tubal microenvironment	Limited	Technically challenging; not routinely collected; research only
Cervical mucus	Speculum exam with swab or aspiration	Minimal (outpatient)	Accessible; reflects lower genital tract; may contain endometrial reflux	Yes	Distal from ovarian pathology; influenced by cervical factors
Cervical swab	Speculum exam with cytobrush	Minimal (outpatient)	Cellular material from cervix; may capture endometrial cells shed retrograde	Yes	Already studied with FTIR [96,97]; indirect reflection of ovarian disease
Vaginal secretions	Swab or lavage	Minimal (self-collection possible)	Easily accessible; contains metabolites and microbiota	Yes	Highly variable; influenced by microbiome, infections, sexual activity
Serum/plasma	Venipuncture	Minimal (routine blood draw)	Systemic reflection; extensively studied	Yes	Dilutes local signals; may not capture ovarian-specific changes
Urine	Voided collection	Non-invasive (self-collection)	Convenient; contains hormone metabolites	Yes	Highly diluted; variable concentration; diurnal variation
Saliva	Passive drool or swab	Non-invasive (self-collection)	Easy to collect; contains hormones and metabolites	Yes	Significant dilution; influenced by oral health, food intake

## Data Availability

No new data were created or analyzed in this study. Data sharing is not applicable to this article.

## Abbreviations

**Abbreviation**	**Full Form**
ART	Assisted Reproductive Technology
ATR	Attenuated Total Reflection
BCAA	Branched-Chain Amino Acid
DOR	Diminished Ovarian Reserve
ELISA	Enzyme-Linked Immunosorbent Assay
FF	Follicular Fluid
FTIR	Fourier-Transform Infrared (Spectroscopy)
HSG	Hysterosalpingography
IUI	Intrauterine Insemination
IVF	In Vitro Fertilization
LDA	Linear Discriminant Analysis
MIS	Minimally Invasive Surgery
MRI	Magnetic Resonance Imaging
PCA	Principal Component Analysis
PCOS	Polycystic Ovary Syndrome
PLS-DA	Partial Least Squares Discriminant Analysis
rASRM	Revised American Society for Reproductive Medicine
REI	Reproductive Endocrinology and Infertility (specialist)
ROS	Reactive Oxygen Species
TVUS	Transvaginal Ultrasonography
WT	Wild Type

## References

[1] Smolarz B., Szyłło K., Romanowicz H. (2021). Endometriosis: Epidemiology, Classification, Pathogenesis, Treatment and Genetics (Review of Literature). Int. J. Mol. Sci..

[2] McLeod B.S., Retzloff M.G. (2010). Epidemiology of endometriosis: An assessment of risk factors. Clin. Obstet. Gynecol..

[3] Parasar P., Ozcan P., Terry K.L. (2017). Endometriosis: Epidemiology, Diagnosis and Clinical Management. Curr. Obstet. Gynecol. Rep..

[4] Szylit N.A., Pasquini Raiza L.C., Schindler Leal A.A., Podgaec S. (2025). Epidemiology with real-world data: Deep endometriosis in women of reproductive age. Einstein.

[5] Varghese N., Shanmugam I., Sivamani H., Durairaj A. (2024). Prevalence and Risk Factors of Endometriosis Among Infertile Women in a Tertiary Care Center in South India. Cureus.

[6] Mishra V.V., Gaddagi R.A., Aggarwal R., Choudhary S., Sharma U., Patel U. (2015). Prevalence; Characteristics and Management of Endometriosis Amongst Infertile Women: A One Year Retrospective Study. J. Clin. Diagn. Res. JCDR.

[7] Nezhat C., Khoyloo F., Tsuei A., Armani E., Page B., Rduch T., Nezhat C. (2024). The Prevalence of Endometriosis in Patients with Unexplained Infertility. J. Clin. Med..

[8] Bulletti C., Coccia M.E., Battistoni S., Borini A. (2010). Endometriosis and infertility. J. Assist. Reprod. Genet..

[9] Bonavina G., Taylor H.S. (2022). Endometriosis-associated infertility: From pathophysiology to tailored treatment. Front. Endocrinol..

[10] Guo J., Wang Y., Chen G. (2024). Causal Relationship Between Endometriosis, Female Infertility, and Primary Ovarian Failure Through Bidirectional Mendelian Randomization. Int. J. Women’s Health.

[11] Somigliana E., Invernici D., Fornelli G., Maria Merli C.E., Vercellini P. (2025). Risk of endometriosis progression in infertile women trying to conceive naturally or using IVF. Hum. Reprod..

[12] Paris K., Aris A. (2010). Endometriosis-associated infertility: A decade’s trend study of women from the Estrie region of Quebec, Canada. Gynecol. Endocrinol. Off. J. Int. Soc. Gynecol. Endocrinol..

[13] Elizur S.E., Mostafa J., Berkowitz E., Orvieto R. (2025). Endometriosis and infertility: Pathophysiology, treatment strategies, and reproductive outcomes. Arch. Gynecol. Obstet..

[14] Khine Y.M., Taniguchi F., Harada T. (2016). Clinical management of endometriosis-associated infertility. Reprod. Med. Biol..

[15] Fadhlaoui A., Feki A. (2014). Endometriosis and Infertility: How and When to Treat?. Front. Surg..

[16] Mayrhofer D., Parry J.P., Hager M., Beitl K., Kurz C., Ott J. (2022). Are the Stage and the Incidental Finding of Endometriosis Associated with Fallopian Tube Occlusion? A Retrospective Cohort Study on Laparoscopic Chromopertubation in Infertile Women. J. Clin. Med..

[17] Ata B., Somigliana E. (2024). Endometriosis, staging, infertility and assisted reproductive technology: Time for a rethink. Reprod. Biomed. Online.

[18] Mohammed Rasheed H.A., Hamid P. (2020). Inflammation to Infertility: Panoramic View on Endometriosis. Cureus.

[19] Tsamantioti E.S., Mahdy H. (2023). Endometriosis. StatPearls [Internet].

[20] Scutiero G., Iannone P., Bernardi G., Bonaccorsi G., Spadaro S., Volta C.A., Greco P., Nappi L. (2017). Oxidative Stress and Endometriosis: A Systematic Review of the Literature. Oxidative Med. Cell. Longev..

[21] Goday A., Méndez M., Cívico Y., Gràcia M., Casals G., Peralta S., Borrás A., Fàbregues F., Agustí I., Barral Y. (2024). Exploring Oxidative Stress in Different Endometriosis Phenoptypes: Insights from Ovarian and Systemic Perspectives by the Study of SIRT3. Int. J. Mol. Sci..

[22] Máté G., Bernstein L.R., Török A.L. (2018). Endometriosis Is a Cause of Infertility. Does Reactive Oxygen Damage to Gametes and Embryos Play a Key Role in the Pathogenesis of Infertility Caused by Endometriosis?. Front. Endocrinol..

[23] Cho Y.J., Kim H.Y. (2018). Oxidative stress and endometriosis. Kosin Med. J..

[24] Lin Y., Chen Y., Chang H., Au H., Tzeng C., Huang Y. (2018). Chronic Niche Inflammation in Endometriosis-Associated Infertility: Current Understanding and Future Therapeutic Strategies. Int. J. Mol. Sci..

[25] Ou Y., Wang H., Zhou C., Chen Y., Lyu J., Feng M., Huang X. (2025). Endometriosis-associated infertility: Multi-omics insights into pathogenesis and precision therapeutics. Front. Endocrinol..

[26] Rathod S., Shanoo A., Acharya N. (2024). Endometriosis: A Comprehensive Exploration of Inflammatory Mechanisms and Fertility Implications. Cureus.

[27] Lamceva J., Uljanovs R., Strumfa I. (2023). The Main Theories on the Pathogenesis of Endometriosis. Int. J. Mol. Sci..

[28] Garrido N., Navarro J., Remohí J., Simón C., Pellicer A. (2000). Follicular hormonal environment and embryo quality in women with endometriosis. Hum. Reprod. Update.

[29] Stilley J.A., Birt J.A., Sharpe-Timms K.L. (2012). Cellular and molecular basis for endometriosis-associated infertility. Cell Tissue Res..

[30] Lin X., Tong X., Zhang Y., Gu W., Huang Q., Zhang Y., Zhuo F., Zhao F., Jin X., Li C. (2022). Decreased Expression of EZH2 in Granulosa Cells Contributes to Endometriosis-Associated Infertility by Targeting IL-1R2. Endocrinology.

[31] Tan Z., Gong X., Wang C.C., Zhang T., Huang J. (2023). Diminished Ovarian Reserve in Endometriosis: Insights from In Vitro, In Vivo, and Human Studies—A Systematic Review. Int. J. Mol. Sci..

[32] Özcan P., Varlı B., Sarıdoğan E., Oral E., Mabrouk M., Usta T., Constantin A.S. (2025). Mechanisms of Endometrioma-Mediated Ovarian Damage: Myths and Facts. J. Clin. Med..

[33] Xu B., Guo N., Zhang X., Shi W., Tong X., Iqbal F., Liu Y. (2015). Oocyte quality is decreased in women with minimal or mild endometriosis. Sci. Rep..

[34] Shebl O., Sifferlinger I., Habelsberger A., Oppelt P., Mayer R.B., Petek E., Ebner T. (2017). Oocyte competence in in vitro fertilization and intracytoplasmic sperm injection patients suffering from endometriosis and its possible association with subsequent treatment outcome: A matched case–control study. Acta Obstet. Gynecol. Scand..

[35] Findikli N., Janssens S., Fasano G., Demeestere I., Fastrez M., Houba C., Delbaere A. (2025). The Effects of Endometriosis on Oocyte and Embryo Quality. J. Clin. Med..

[36] Liao L., Pan Z., Li Y. (2025). Endometriosis as a risk factor: Impact on IVF outcomes and reproductive parameters: A systematic review and meta-analysis. Arch. Gynecol. Obstet..

[37] Simopoulou M., Rapani A., Grigoriadis S., Pantou A., Tsioulou P., Maziotis E., Tzanakaki D., Triantafyllidou O., Kalampokas T., Siristatidis C. (2021). Getting to Know Endometriosis-Related Infertility Better: A Review on How Endometriosis Affects Oocyte Quality and Embryo Development. Biomedicines.

[38] Fan Y., Yang Q., Lin Y., Fu X., Shu J. (2025). The effect of endometriosis on oocyte quality: Mechanisms, diagnosis and treatment. Arch. Gynecol. Obstet..

[39] Sanchez A.M., Vanni V.S., Bartiromo L., Papaleo E., Zilberberg E., Candiani M., Orvieto R., Viganò P. (2017). Is the oocyte quality affected by endometriosis? A review of the literature. J. Ovarian Res..

[40] Younis J.S. (2022). Is Oocyte Quality Impaired in Cases with Ovarian Endometriosis? A Second Look into the Clinical Setting. Front. Endocrinol..

[41] Revelli A., Piane L.D., Casano S., Molinari E., Massobrio M., Rinaudo P. (2009). Follicular fluid content and oocyte quality: From single biochemical markers to metabolomics. Reprod. Biol. Endocrinol..

[42] Pan Y., Pan C., Zhang C. (2024). Unraveling the complexity of follicular fluid: Insights into its composition, function, and clinical implications. J. Ovarian Res..

[43] Brinca A.T., Ramalhinho A.C., Sousa Â., Oliani A.H., Breitenfeld L., Passarinha L.A., Gallardo E. (2022). Follicular Fluid: A Powerful Tool for the Understanding and Diagnosis of Polycystic Ovary Syndrome. Biomedicines.

[44] Emori M.M., Drapkin R. (2014). The hormonal composition of follicular fluid and its implications for ovarian cancer pathogenesis. Reprod. Biol. Endocrinol..

[45] Yu L., Liu M., Xu S., Wang Z., Liu T., Zhou J., Zhang D., Dong X., Pan B., Wang B. (2022). Follicular fluid steroid and gonadotropic hormone levels and mitochondrial function from exosomes predict embryonic development. Front. Endocrinol..

[46] Zhang X., Wang T., Song J., Deng J., Sun Z. (2020). Study on follicular fluid metabolomics components at different ages based on lipid metabolism. Reprod. Biol. Endocrinol..

[47] Lai D., Xu M., Zhang Q., Chen Y., Li T., Wang Q., Gao Y., Wei C. (2015). Identification and characterization of epithelial cells derived from human ovarian follicular fluid. Stem Cell Res. Ther..

[48] Collodel G., Gambera L., Stendardi A., Nerucci F., Signorini C., Pisani C., Marcheselli M., Vellucci F.L., Pizzasegale S.E., Micheli L. (2023). Follicular Fluid Components in Reduced Ovarian Reserve, Endometriosis, and Idiopathic Infertility. Int. J. Mol. Sci..

[49] Moreira M.V., Vale-Fernandes E., Albergaria I.C., Alves M.G., Monteiro M.P. (2023). Follicular fluid composition and reproductive outcomes of women with polycystic ovary syndrome undergoing in vitro fertilization: A systematic review. Rev. Endocr. Metab. Disord..

[50] von Mengden L., Klamt F., Smitz J. (2020). Redox biology of human cumulus cells: Basic concepts, impact on oocyte quality, and potential clinical use. Antioxid. Redox Signal..

[51] Da Broi M.G., I Giorgi V.S., Wang F., Keefe D.L., Albertini D., Navarro P.A. (2018). Influence of follicular fluid and cumulus cells on oocyte quality: Clinical implications. J. Assist. Reprod. Genet..

[52] Warzych E., Lipinska P. (2020). Energy metabolism of follicular environment during oocyte growth and maturation. J. Reprod. Dev..

[53] Clark H.M., Stokes A.E., Edwards J.L., Payton R.R., Schrick F.N., Campagna S.R., Sarumi Q., Hessock E.A., Roberts S.R., Azaridolatabad N. (2024). Impact of preovulatory follicle maturity on oocyte metabolism and embryo development. PNAS Nexus.

[54] Lipinska P., Smits K., Soom A.V., Pavani K.C., Warzych E. (2025). Follicular-fluid extracellular vesicles support energy metabolism of bovine oocytes, improving blastocyst development and quality. Biol. Reprod..

[55] Kassem A., Abbas L., Coutinho O., Opara S., Najaf H., Kasperek D., Pokhrel K., Li X., Tiquia-Arashiro S. (2023). Applications of Fourier Transform-Infrared spectroscopy in microbial cell biology and environmental microbiology: Advances, challenges, and future perspectives. Front. Microbiol..

[56] Berthomieu C., Hienerwadel R. (2009). Fourier transform infrared (FTIR) spectroscopy. Photosynth. Res..

[57] Fadlelmoula A., Pinho D., Carvalho V.H., Catarino S.O., Minas G. (2022). Fourier Transform Infrared (FTIR) Spectroscopy to Analyse Human Blood over the Last 20 Years: A Review towards Lab-on-a-Chip Devices. Micromachines.

[58] Fernández-Galiana Á., Bibikova O., Vilms Pedersen S., Stevens M.M. (2024). Fundamentals and Applications of Raman-Based Techniques for the Design and Development of Active Biomedical Materials. Adv. Mater..

[59] Cordero E., Latka I., Matthäus C., Schie I., Popp J. (2018). In-vivo Raman spectroscopy: From basics to applications. J. Biomed. Opt..

[60] Jones R.R., Hooper D.C., Zhang L., Wolverson D., Valev V.K. (2019). Raman Techniques: Fundamentals and Frontiers. Nanoscale Res. Lett..

[61] Saletnik A., Saletnik B., Puchalski C. (2021). Overview of Popular Techniques of Raman Spectroscopy and Their Potential in the Study of Plant Tissues. Molecules.

[62] Orlando A., Franceschini F., Muscas C., Pidkova S., Bartoli M., Rovere M., Tagliaferro A. (2021). A Comprehensive Review on Raman Spectroscopy Applications. Chemosensors.

[63] Berna F., Gilbert A.S. (2017). Fourier Transform Infrared Spectroscopy (FTIR). Encyclopedia of Geoarchaeology.

[64] Al-Kelani M., Buthelezi N. (2024). Advancements in medical research: Exploring Fourier Transform Infrared (FTIR) spectroscopy for tissue, cell, and hair sample analysis. Ski. Res. Technol..

[65] Kaur M., Singh S., Kaur A. (2024). Structural changes in amide I and amide II regions of PCOS women analyzed by ATR-FTIR spectroscopy. Heliyon.

[66] Olcha P., Paja W., Kępski M., Pancerz K., Klebowski B., Nowakowski Ł., Gałczyński K., Depciuch J. (2025). Biochemical Heterogeneity of Endometriosis Phenotypes Revealed by FTIR Analysis. J. Biophotonics.

[67] Xiong Y., Zhu H., Shi R., Wu Y., Fan Y., Jin L. (2024). Regulation of glucose metabolism: Effects on oocyte, preimplantation embryo, assisted reproductive technology and embryonic stem cell. Heliyon.

[68] Cheung K.T., Trevisan J., Kelly J.G., Ashton K.M., Stringfellow H.F., Taylor S.E., Singh M.N., Martin-Hirsch P.L., Martin F.L. (2011). Fourier-transform infrared spectroscopy discriminates a spectral signature of endometriosis independent of inter-individual variation. Analyst.

[69] Olcha P., Paja W., Kępski M., Pancerz K., Klebowski B., Nowakowski Ł., Gałczyński K., Depciuch J. (2026). Detection of peritoneal, ovarian, and bowel endometriosis using FTIR spectroscopy and machine learning. Spectrochim. Acta Part A Mol. Biomol. Spectrosc..

[70] Dietzek B., Cialla D., Schmitt M., Popp J., Dieing T., Hollricher O., Toporski J. (2010). Introduction to the Fundamentals of Raman Spectroscopy. Confocal Raman Microscopy.

[71] Guleken Z., Bulut H., Bulut B., Paja W., Parlinska-Wojtan M., Depciuch J. (2022). Correlation between endometriomas volume and Raman spectra. Attempting to use Raman spectroscopy in the diagnosis of endometrioma. Spectrochim. Acta Part A Mol. Biomol. Spectrosc..

[72] Notarstefano V., Gioacchini G., Byrne H.J., Zacà C., Sereni E., Vaccari L., Borini A., Carnevali O., Giorgini E. (2019). Vibrational characterization of granulosa cells from patients affected by unilateral ovarian endometriosis: New insights from infrared and Raman microspectroscopy. Spectrochim. Acta Part A Mol. Biomol. Spectrosc..

[73] Parlatan U., Inanc M.T., Ozgor B.Y., Oral E., Bastu E., Unlu M.B., Basar G. (2019). Raman spectroscopy as a non-invasive diagnostic technique for endometriosis. Sci. Rep..

[74] Bartnik K., Koba M., Śmietana M. (2023). Advancements in optical fiber sensors for in vivo applications—A review of sensors tested on living organisms. Measurement.

[75] Kluz-Barlowska T., Paja W., Pancerz K., Miziak P., Cebulski J., Depciuch J. (2024). FT-Raman and FTIR spectroscopy as a tools showing marker of platinum-resistant phenomena in women suffering from ovarian cancer. Sci. Rep..

[76] Bridelli M.G. (2017). Fourier Transform Infrared Spectroscopy in the Study of Hydrated Biological Macromolecules. Fourier Transforms-High-Tech Application and Current Trends.

[77] Chan K.L.A., Kazarian S.G. (2016). Attenuated total reflection Fourier-transform infrared (ATR-FTIR) imaging of tissues and live cells. Chem. Soc. Rev..

[78] Wolkers W.F., Bochicchio A., Selvaggi G., Hoekstra F.A. (1998). Fourier transform infrared microspectroscopy detects changes in protein secondary structure associated with desiccation tolerance in developing maize embryos. Plant Physiol..

[79] Prestrelski S., Tedeschi N., Arakawa T., Carpenter J. (1993). Dehydration-induced conformational transitions in proteins and their inhibition by stabilizers. Biophys. J..

[80] Bassan P., Mellor J., Shapiro J., Williams K.J., Lisanti M.P., Gardner P. (2014). Transmission FT-IR chemical imaging on glass substrates: Applications in infrared spectral histopathology. Anal. Chem..

[81] Ramoji A., Galler K., Glaser U., Henkel T., Mayer G., Dellith J., Bauer M., Popp J., Neugebauer U. (2016). Characterization of different substrates for Raman spectroscopic imaging of eukaryotic cells. J. Raman Spectrosc..

[82] Tian Z.Q., Ren B., Wu D.Y. (2002). Surface-enhanced Raman scattering: From noble to transition metals and from rough surfaces to ordered nanostructures. J. Phys. Chem. B.

[83] Cutshaw G., Uthaman S., Hassan N., Kothadiya S., Wen X., Bardhan R. (2023). The Emerging Role of Raman Spectroscopy as an Omics Approach for Metabolic Profiling and Biomarker Detection towards Precision Medicine. Chem. Rev..

[84] Lo Turco E.G., Cordeiro F.B., Lopes P.H., Gozzo F.C., Pilau E.J., Soler T.B., da Silva B.F., Del Giudice P.T., Bertolla R.P., Fraietta R. (2013). Proteomic analysis of follicular fluid from women with and without endometriosis: New therapeutic targets and biomarkers. Mol. Reprod. Dev..

[85] Cao X.L., Song J.Y., Sun Z.G. (2022). Quantitative label-free proteomic analysis of human follicle fluid to identify novel candidate protein biomarker for endometriosis-associated infertility. J. Proteom..

[86] Kurdi C., Hesszenberger D., Csabai D., Lajtai A., Lakatos Á., Gödöny K., Mauchart P., Várnagy Á., Kovács G.L., Kőszegi T. (2025). Follicular Fluid Amino Acid Alterations in Endometriosis: Evidence for Oxidative Stress and Metabolic Dysregulation. Biomedicines.

[87] Kobayashi H., Imanaka S. (2024). Recent progress in metabolomics for analyzing common infertility conditions that affect ovarian function. Reprod. Med. Biol..

[88] Karaer A., Tuncay G., Mumcu A., Dogan B. (2019). Metabolomics analysis of follicular fluid in women with ovarian endometriosis undergoing in vitro fertilization. Syst. Biol. Reprod. Med..

[89] Ortiz C.N., Torres-Reverón A., Appleyard C.B. (2021). Metabolomics in endometriosis: Challenges and perspectives for future studies. Reprod. Fertil..

[90] Przewocki J., Łukaszuk A., Jakiel G., Wocławek-Potocka I., Kłosińska K., Olszewska J., Łukaszuk K. (2024). Proteomic Analysis of Follicular Fluid in Polycystic Ovary Syndrome: Insights into Protein Composition and Metabolic Pathway Alterations. Int. J. Mol. Sci..

[91] Zhang Y., He C., He Y., Zhu Z. (2024). Follicular Fluid Metabolomics: Tool for Predicting IVF Outcomes of Different Infertility Causes. Reprod. Sci..

[92] Marianna S., Alessia P., Susan C., Francesca C., Angela S., Francesca C., Antonella N., Patrizia I., Nicola C., Emilio C. (2017). Metabolomic profiling and biochemical evaluation of the follicular fluid of endometriosis patients. Mol. Biosyst..

[93] Yu P., Chen D., Han D., Jin X., Li Y., Wei F., Zhang Y. (2026). Metabolomic profiling of follicular fluid reveals unique pathways in endometriosis and infertility etiologies: A pilot study. PeerJ.

[94] Guo H., Zhu Q., Gao H., Lyu Q., Chai W., Wu L., Li B. (2023). Metabolomics analysis of follicular fluid in ovarian endometriosis women receiving progestin-primed ovary stimulation protocol for in vitro fertilization. Sci. Rep..

[95] Li J., Zhang Z., Wei Y., Zhu P., Yin T., Wan Q. (2023). Metabonomic analysis of follicular fluid in patients with diminished ovarian reserve. Front. Endocrinol..

[96] Karakaşlı A., Görkem Ü., Toğrul C., Yıldırım E., Köse D.A., Yurdakul Ö. (2025). Non-invasive diagnosis of endometrioma through cervical swabs using Fourier transform infrared spectroscopy. Turk. J. Obstet. Gynecol..

[97] Bozdag G., Igci N., Calis P., Ayhan B., Ozel Demiralp D., Mumusoglu S., Yarali H. (2019). Examination of cervical swabs of patients with endometriosis using Fourier transform infrared spectroscopy. Arch. Gynecol. Obstet..

[98] Gioacchini G., Sereni E., Zacà C., Giorgini E., Notarstefano V., Vaccari L., Borini A. (2015). Could the unilateral ovarian endometriosis affect the contralateral ovary? new insights from Fourier Transform infrared (FTIR) spectroscopy. Fertil. Steril..

[99] Thomas N., Goodacre R., Timmins E.M., Gaudoin M., Fleming R. (2000). Fourier transform infrared spectroscopy of follicular fluids from large and small antral follicles. Hum. Reprod..

[100] Huang X., Hong L., Wu Y., Chen M., Kong P., Ruan J., Teng X., Wei Z. (2021). Raman Spectrum of Follicular Fluid: A Potential Biomarker for Oocyte Developmental Competence in Polycystic Ovary Syndrome. Front. Cell Dev. Biol..

[101] Zhang X., Liang B., Zhang J., Hao X., Xu X., Chang H.M., Leung P.C.K., Tan J. (2021). Raman spectroscopy of follicular fluid and plasma with machine-learning algorithms for polycystic ovary syndrome screening. Mol. Cell. Endocrinol..

[102] Depciuch J., Paja W., Pancerz K., Uzun Ö., Bulut H., Tarhan N., Guleken Z. (2023). Analysis of follicular fluid and serum markers of oxidative stress in women with unexplained infertility by Raman and machine learning methods. J. Raman Spectrosc..

[103] Barnas E., Skret A., Kaznowska E., Depciuch J., Szmuc K., Łach K., Cebulski J. (2019). Simultaneous FTIR and Raman Spectroscopy in Endometrial Atypical Hyperplasia and Cancer. Int. J. Mol. Sci..

